# Development and validation of a high-throughput calcium mobilization assay for the orphan receptor GPR88

**DOI:** 10.1186/s12929-017-0330-3

**Published:** 2017-03-27

**Authors:** Ann M. Decker, Elaine A. Gay, Kelly M. Mathews, Taylor C. Rosa, Tiffany L. Langston, Rangan Maitra, Chunyang Jin

**Affiliations:** Center for Drug Discovery, Research Triangle Institute, Post Office Box 12194, Research Triangle Park, NC 27709 USA

**Keywords:** GPR88, Gα_qi5_, 2-PCCA, High-throughput screen, Calcium mobilization

## Abstract

**Background:**

GPR88 is an orphan G protein-coupled receptor highly expressed in the striatum and is implicated in basal ganglia-associated disorders. However, the receptor functions of GPR88 are still largely unknown due to the lack of potent and selective ligands appropriate for central nervous system investigation. Development of a high-throughput screening assay for GPR88 should facilitate the discovery of novel ligands to probe GPR88 functions.

**Methods:**

In this paper, we describe the development of a CHO-Gα_qi5_-GPR88 cell-based calcium mobilization assay. The assay takes advantage of functional coupling of GPR88 with the promiscuous Gα_qi5_ protein and consequent mobilization of intracellular calcium, which can be measured in a 384-well format with a Fluorescent Imaging Plate Reader.

**Results:**

The CHO-Gα_qi5_-GPR88 cell-based calcium mobilization assay was validated by the structure-activity relationship study of known GPR88 agonist (1*R*,2*R*)-2-PCCA analogues. The assay was automated and miniaturized to a 384-well format, and was deemed robust and reproducible with a Z’-factor of 0.72 and tolerated dimethyl sulfoxide to a final concentration of 2%. Screening a pilot neurotransmitter library consisting of 228 compounds yielded 10 hits, but none of the hits were confirmed as GPR88 agonists in follow-up assays.

**Conclusions:**

We have developed a high-throughput calcium mobilization assay for the orphan receptor GPR88. This calcium mobilization assay can be used to identify several different types of GPR88 ligands including agonists, competitive and noncompetitive antagonists, inverse agonists, and allosteric modulators. These ligands will serve as valuable tools to probe signaling mechanisms and in vivo functions of GPR88, and could expedite development of novel therapies for diseases potentially mediated by GPR88.

**Electronic supplementary material:**

The online version of this article (doi:10.1186/s12929-017-0330-3) contains supplementary material, which is available to authorized users.

## Background

Basal ganglia dysfunction is implicated in a number of neurological and psychiatric disorders including Parkinson’s and Huntington’s diseases, schizophrenia, bipolar disorder, and drug abuse [1]. Although dopaminergic and glutamatergic systems are often used as drug targets to treat basal ganglia-associated disorders [2, 3], considerable efforts have also focused on identifying alternative mechanisms and new candidate proteins that regulate basal ganglia functions, which may lead to new drug discovery opportunities [4, 5]. The orphan G protein-coupled receptor (GPCR) GPR88 is a potential candidate for such efforts.

GPR88 is highly expressed in striatal GABAergic medium spiny neurons (MSNs), which are involved in both the striatonigral and striatopallidal pathways [6–8]. GPR88 is also expressed in other brain regions including the cerebral cortex, amygdala, and hypothalamus [9–11]. Multiple lines of evidence obtained from GPR88-knockout studies suggest that GPR88 plays an important role in regulating striatal functions and is implicated in basal ganglia-associated disorders. In past studies, GPR88-knockout mice demonstrated disrupted prepulse inhibition of startle response, a phenotype of schizophrenia, and exhibited D_2_ receptor hypersensitivity, as evidenced by increased sensitivity to amphetamine-stimulated locomotor activity and to apomorphine-induced climbing and stereotypy [12]. In a different study, GPR88-deficient mice exhibited increased locomotion, poor motor coordination and impaired cue-based learning, which could be normalized by targeted viral expression of the receptor in striatal MSNs [8]. A recent study also linked GPR88 to anxiety disorders, as the GPR88-knockout mice exhibited low anxiety compared to wild-type animals [13].

We previously reported that a synthetic small molecule (1*R*,2*R*)-2-PCCA [(1*R*,2*R*)-2-(pyridin-2-yl)cyclopropanecarboxylic acid ((2*S*,3*S*)-2-amino-3-methylpentyl)-(4’-propylbiphenyl-4-yl)amide] was able to modulate GPR88 activity. This agonist inhibited isoproterenol-stimulated cAMP accumulation with an EC_50_ = 603 nM in a GloSensor cAMP assay using stable HEK293-GPR88-pGloSensor22F cells, suggesting GPR88 is coupled to Gα_i_ proteins [14]. Recently, we showed that (1*R*,2*R*)-2-PCCA had an EC_50_ = 56 nM, in line with the EC_50_ value reported by Bi et al. [15], in a LANCE^®^ Ultra cAMP assay using stable PPLS-HA-GPR88 CHO cells [16]. The discrepancy between the EC_50_ values of (1*R*,2*R*)-2-PCCA is possibly due to the sensitivity of the different assay systems. Another type of compound structurally related to 2-PCCA, represented by 2-AMPP [(2*S*)-*N*-((1*R*)-2-amino-1-(4-(2-methylpentyloxy)phenyl)ethyl)-2-phenylpropanamide], was also shown to activate GPR88 [17, 18]. Although (1*R*,2*R*)-2-PCCA is useful in the characterization of in vitro signaling pathways of GPR88, it may not be as useful as an in vivo probe due to its high lipophilicity (cLogP 6.19) and reports that it is a P-glycoprotein substrate, both of which indicate that the compound is expected to have poor brain penetration [15]. Clearly, there is an unmet need for GPR88 in vivo probes appropriate for CNS investigation. Structurally different chemotypes with better drug-like characteristics showing potent and selective GPR88 agonism/antagonism will be particularly helpful in this regard. Development of a high-throughput screening (HTS) assay for GPR88 should facilitate the discovery of novel ligands as in vivo probes of GPR88 function. Herein we report the development of a calcium mobilization based HTS assay for GPR88, which was validated by the structure-activity relationship (SAR) study of a small group of 2-PCCA analogues.

## Methods

### Drugs

(1*R*,2*R*)-2-PCCA (**1a**, Table 1) and analogues (**1b-d**, **2a-c** and **3**) were synthesized following methods detailed in our early work [14, 16]. All target compounds were characterized by NMR and HRMS analyses, and the spectral data were in agreement with the assigned structures (see Additional file 1).

**Table 1:** EC_50_ determination of (1*R*,2*R*)-2-PCCA analogues


Compd^a^	X	R^1^	R^2^	pEC_50_ (EC_50_, nM)^b^
(1*R*,2*R*)-2-PCCA (**1a**)	N	Propyl		6.33 ± 0.06 (468)
**1b**	C	Propyl		5.90 ± 0.08 (1260)
**1c**	C	Cyclohexyl		6.10 ± 0.04 (794)
**1d**	C	CONH_2_		NA^c^
**2a**			(*S*)-NH_2_	5.88 ± 0.03 (1320)
**2b**			(*R*)-NH_2_	5.58 ± 0.04 (2630)
**2c**			H	NA^c^
**3**				NA^c^

^a^All compounds were tested as the HCl salt

^b^pEC_50_ values are means ± standard error of at least three independent experiments performed in duplicate

^c^EC_50_ > 10 μM, tested in two independent experiments performed in duplicate

### Cell culture and molecular biology

Cell culture materials were purchased from Fisher SSI. Lipofectamine LTX with PLUS reagent was purchased from ThermoFisher Scientific. FuGene was purchased from Promega. Gα_qi5_-CHO stable cells were purchased from GenScript (Piscataway, NJ) and were cultured in Ham’s F12 supplemented with 10% fetal bovine serum (FBS), 100 units each of penicillin and streptomycin (P/S), and 100 μg/mL Hygromycin B. An expression plasmid containing a pre-prolactin leader sequence (PPLS) and an influenza hemagglutanin (HA) tag fused in frame upstream of the human GPR88 cDNA was prepared in a modified pcDNA3.1+ mammalian expression vector by GenScript. Midi-prep DNA was prepared using a Qiagen HiSpeed Plasmid Midi Kit and the PPLS-HA-GPR88 construct was verified by sequencing.

### Transient transfections

CHO-RD-HGA16 (Molecular Devices) cells stably expressing the Gα_q16_ protein were plated into 6-well dishes at 200,000 cells/well and incubated at 37 °C, 5% CO_2_ overnight. The next day, cells were transfected with an expression vector containing human GPR88 cDNA (1000 ng) using FuGene. The next day, cells were lifted and plated into 96-well black-walled clear bottom assay plates and the calcium mobilization assay (as described in the 96-well methods) was run the following day using 10 μM final (1*R*,2*R*)-2-PCCA to stimulate the cells. Gα_qi5_-CHO cells were plated into 6-well dishes at 250,000 cells/well and incubated at 37 °C, 5% CO_2_ overnight. The next day, cells were transfected with an expression vector containing human PPLS-HA-GPR88 cDNA (2500 ng) using Lipofectamine LTX with PLUS reagent. Transfected cells were plated and assayed as described for the Gα_q16_ cells.

### Creation of stable PPLS-HA-GPR88-Gα_qi5_-CHO cell line

Gα_qi5_-CHO cells were transfected with PPLS-HA-GPR88 using Lipofectamine LTX with PLUS reagent. Transfected cells were plated into 10 cm^2^ dishes 24 h post transfection and were selected using Geneticin. After 2 weeks, individual clones were selected, grown to confluence, and screened in a functional 96-well calcium mobilization assay that measured response to (1*R*,2*R*)-2-PCCA (10 μM final). Concentration-response curves of (1*R*,2*R*)-2-PCCA were run in clones that showed a high response to the agonist in the screen. The final working clone with the most potent (1*R*,2*R*)-2-PCCA response and the largest signal window (EC_50_ = 468 nM; signal window = 6000 RFU) was chosen for all subsequent studies.

### 96-well calcium mobilization assay

The day before the assay, stable PPLS-HA-GPR88-Gα_qi5_-CHO cells were plated into 96-well black-walled assay plates at 30,000 cells/well (100 μL per well) in Ham’s F12 supplemented with 10% FBS and 100 units each of P/S. The cells were incubated overnight at 37 °C, 5% CO_2_. Prior to the assay, Calcium 5 dye (Bulk Kit, Molecular Devices) was reconstituted according to the manufacturer’s instructions. The reconstituted dye was diluted 1:40 in pre-warmed (37 °C) assay buffer (1X HBSS, 20 mM HEPES, 2.5 mM probenecid, pH 7.4 at 37 °C). Medium was removed from the plate, and the cells were gently washed with 100 μL of pre-warmed (37 °C) assay buffer. The diluted Calcium 5 dye (200 μL) was added and the cells were incubated for 45 min at 37 °C, 5% CO_2_. During the incubation, serial dilutions of (1*R*,2*R*)-2-PCCA were prepared in 0.25% BSA/1% DMSO/assay buffer, aliquoted into 96-well polypropylene plates, and warmed to 37 °C. After the dye-loading incubation period, the cells were pretreated with 25 μL of 2.25% BSA/9% DMSO/assay buffer and incubated for 15 min at 37 °C. After the pretreatment incubation period, the plate was read with a FlexStation II (Molecular Devices). Calcium-mediated changes in fluorescence were monitored every 1.52 s over a 60 s time period, with the FlexStation II adding 25 μL of test compound dilutions at the 19 s time point (excitation at 485 nm, detection at 525 nm). Peak kinetic reduction (SoftMax, Molecular Devices) relative fluorescent units (RFU) were plotted against the log of compound concentration using nonlinear regression analysis to generate EC_50_ values (GraphPad Prism 6.0, GraphPad Software, Inc., San Diego, CA).

### Tolerance to DMSO

Tolerance to DMSO was determined by evaluating the (1*R*,2*R*)-2-PCCA response at multiple final concentrations of DMSO (1, 1.5, 2, 2.5, 3, 3.5%) in the functional calcium mobilization assay.

### 384-well Z’-factor determination and no-wash calcium mobilization protocol

The stable PPLS-HA-GPR88-Gα_qi5_-CHO cells were plated in 30 μL/well volume at 9000 cells/well in Ham’s F12 medium supplemented with 1% FBS and 100 units each of P/S in 384-well Greiner μClear® black walled microplates using a MicroFlo™ Select dispenser fitted with a 5 μL cassette (BioTek). The plated cells were incubated overnight at 37 °C, 5% CO_2_. The next day, Calcium 5 dye (Bulk Kit, Molecular Devices) was reconstituted according to the manufacturer’s instructions. The reconstituted dye was diluted 1:20 in pre-warmed (37 °C) assay buffer (1X HBSS, 20 mM HEPES, 2.5 mM probenecid, pH 7.4 at 37 °C) and 30 μL was add to the plate (no-wash protocol), which was then incubated for 45 min at 37 °C, 5% CO_2_. During the incubation, 10 μM of (1*R*,2*R*)-2-PCCA was prepared at 10x the desired final concentration in 0.25% BSA/2% DMSO/assay buffer, aliquoted into 384-well polypropylene plates, and warmed to 37 °C. Using the Fluorescent Imaging Plate Reader (FLIPR) Tetra (Molecular Devices), the dye loaded plate was pretreated with 8.5 μL 2% BSA/16% DMSO/assay buffer and incubated for 15 min at 37 °C, 5% CO_2_. After this incubation period, the plate was read with the Tetra. Calcium-mediated changes in fluorescence were monitored every 1 s over a 60 s time period, with the Tetra adding 8.5 μL of (1*R*,2*R*)-2-PCCA or 0.25% BSA/2% DMSO/assay buffer at the 10 s time point (excitation at 470-495 nm, detection at 515-575 nm). Data was collected in ScreenWorks version 3.2 (Molecular Devices). The bias was subtracted on image 8 and the maximum-minimum relative fluorescent units (RFU) statistic was used to calculate the Z’-factor [19] using the equation ${Z}^{\prime }=1 - \left(\frac{3 \times \left({\sigma}_{agonist} + {\sigma}_{control}\right)}{\left|{\mu}_{agonist} - {\mu}_{control}\right|}\right)$ where σ is the mean, μ is the standard deviation, agonist is the signal produced by (1*R*,2*R*)-2-PCCA and control is the vehicle (0.25% BSA/2% DMSO/assay buffer).

### HTS of a pilot neurotransmitter library

The PPLS-HA-GPR88-Gα_qi5_-CHO cells were used to screen a small neurotransmitter library purchased from Enzo Life Sciences (cat. BML-2811, BML-2813 and BML-2819; 228 compounds, Farmingdale, NY). The mother plates from this library were compressed and replicated into 384-well polypropylene daughter plates containing 1 μL of compound at 10 mM in 100% DMSO. All plates were heat sealed with an Agilent Technologies PlateLoc sealer and were stored at -20 °C. Columns 1-2 and 23-24 were left empty during the compression/replication process for assay controls. All compound library plates were barcoded for tracking purposes; plate barcodes link compound identities in RTI International’s institutional database, ChemCart (DeltaSoft).

On each day of the primary screen, the daughter plates with 1 μL of compound were thawed, spun at 1000 rpm for 3 min, unsealed, diluted with 49 μL 0.25% BSA/assay buffer, and mixed using a Biomek NX fitted with a 384 multi-channel dispense head (Beckman Coulter). Assay controls, consisting of a concentration-response curve of (1*R*,2*R*)-2-PCCA, 10 μM final (1*R*,2*R*)-2-PCCA, and 0.25% BSA/2% DMSO/assay buffer, were added to columns 1, 2, 23, and 24 of each plate and each plate was kept at 37 °C. The assay was run and the data were collected as described in the 384-well methods section. The raw RFUs for each compound were converted to % (1*R*,2*R*)-2-PCCA E_max_ using the equation: $\%{E}_{max} = \left(\frac{RF{ U}_{cmpd}}{RF{ U}_{PCCA}}\right) \times 100$

## Results

### Creation of stable PPLS-HA-GPR88-Gα_qi5_-CHO cell line and development of calcium mobilization assay for GPR88

**Fig. 1:**
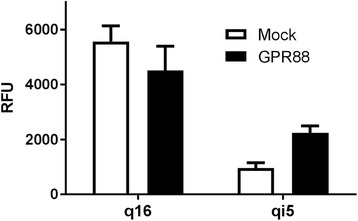
Response of GPR88-Gα_q16_ and GPR88-Gα_qi5_ transients to (1*R*,2*R*)-2-PCCA. Mock cells and GPR88 transfected cells were stimulated with 10 μM final (1*R*,2*R*)-2-PCCA 48 h after transfection in a calcium mobilization assay. Representative data are shown and each bar is the mean ± SD of duplicate determinations

Recently, we have demonstrated that GPR88 couples to Gα_i_ and functionally inhibits cAMP production upon activation by the agonist (1*R*,2*R*)-2-PCCA [14]. In addition, 2-PCCA does not induce calcium mobilization in GPR88 cells, indicating that GPR88 is likely not coupled to Gα_q_ proteins. With these factors in mind, a heterologous system was designed in which GPR88 could couple to the promiscuous Gα_q_ subunit in CHO cells to induce intracellular calcium mobilization. To determine which promiscuous G protein would be most effective for GPR88, we transiently transfected Gα_q16_ and Gα_qi5_ cells with GPR88 and tested the response to 10 μM (1*R*,2*R*)-2-PCCA in a calcium mobilization assay (Fig. 1). In the Gα_q16_-GPR88 transients, (1*R*,2*R*)-2-PCCA produced a large calcium mobilization signal; however, a slightly stronger signal was also produced in the Gα_q16_ mock cells, indicating that (1*R*,2*R*)-2-PCCA does not elicit a GPR88-specific response in the engineered Gα_q16_ system. In the Gα_qi5_-GPR88 transients, (1*R*,2*R*)-2-PCCA produced a 2.3-fold specific response compared to the Gα_qi5_ mock cells, thus suggesting that this engineered system is suitable for stable cell line creation to monitor GPR88 activity.

**Fig. 2:**
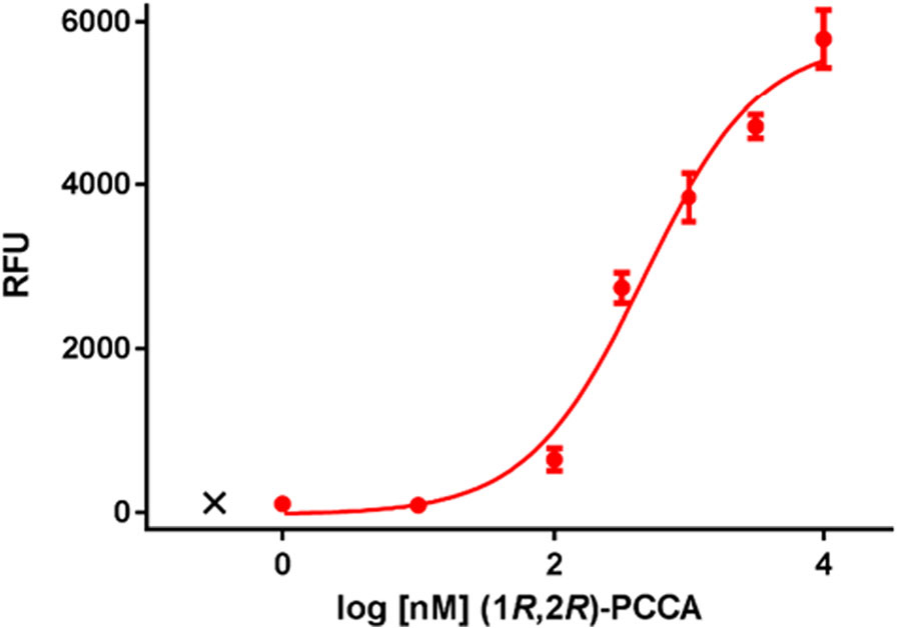
Concentration-response curve of (1*R*,2*R*)-2-PCCA in PPLS-HA-GPR88-Gα_qi5_-CHO cells. Data points are means ± SEM of three independent experiments run in duplicate in the working clone. EC_50_ = 468 nM

Gα_qi5_-CHO cells were transfected with a mammalian expression plasmid containing the PPLS and HA epitope tag fused in frame upstream of GPR88. The PPLS was used to help promote transport of GPR88 to the plasma membrane [20]. Transfected cells were kept in antibiotic selection for 2 weeks, after which individual clones were selected and tested in a functional calcium mobilization assay. Stimulation with 10 μM final (1*R*,2*R*)-2-PCCA resulted in a calcium mobilization response mediated through coupling of GPR88 with Gα_qi5_ as expected. After evaluating 82 clones, a single clone with the most potent EC_50_ value (468 nM) and the largest signal window (6000 RFU) was selected as our working clone (Fig. 2).

### Effect of solvent on assay performance

**Fig. 3:**
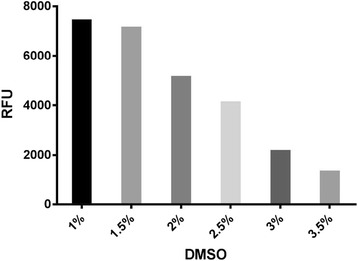
DMSO sensitivity. The effect of DMSO on the assay’s signal window was evaluated by stimulating cells with 10 μM final (1*R*,2*R*)-2-PCCA containing varied DMSO concentrations (1, 1.5, 2, 2.5, 3, 3.5% final). The assay performance is not critically affected up to 1.5% DMSO. Representative data from one experiment are shown

The effect of DMSO on the assay’s performance was evaluated at multiple final concentrations (1, 1.5, 2, 2.5, 3, 3.5%). This study used 1% DMSO as the lowest concentration because most compound libraries are supplied at 10 mM in DMSO and screened at 10 μM final. As shown in Fig. 3, the assay’s performance was not critically affected up to 1.5% final DMSO concentration, indicating that a final screening concentration of 10 μM is achievable with this assay. At 2% final DMSO, the maximum response to (1*R*,2*R*)-2-PCCA decreased by 1.4-fold, but was still appropriate for screening, indicating that compounds can be screened at a final concentration of 20 μM. Starting at 2.5% final DMSO, the response was adversely affected due to a decline in cell health after being exposed to higher concentrations of DMSO.

### Assay validation

To further validate the function of GPR88 in our stable cell line, we tested a series of (1*R*,2*R*)-2-PCCA analogues in the calcium mobilization assay. The results are reported in Table 1. (1*R*,2*R*)-2-PCCA (**1a**) had an EC_50_ = 468 nM, comparable to our previously reported EC_50_ of 603 nM in a GloSensor cAMP assay using stable GPR88-pGloSensor22F cells [14]. In general, the SAR trends observed in the calcium mobilization assay are in good agreement with the results previously reported using cAMP assays [14–16]. These comparison results further validate the GPR88-Gα_qi5_ calcium assay as being capable of measuring activity of GPR88 agonists.

### HTS assay development

**Fig. 4:**
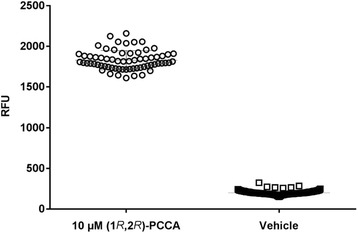
Z’-factor of 384-well assay. The Z’-factor was determined for the automated 384-well calcium mobilization assay by using (1*R*,2*R*)-2-PCCA (○) as the positive control and 2% final DMSO as the negative control (□) on at least two plates each day for four individual days. Representative data are shown and each data point on the graph is the response from a single well on one plate

To make our 96-well calcium mobilization assay more amenable to HTS, we automated and miniaturized the assay to 384-well. Multiple cell densities were tested in 384-well format to determine the optimal number of cells to use for screening, which was determined to be 9000 cells/well based on the assay’s signal window and (1*R*,2*R*)-2-PCCA EC_50_ value. Automated cell dispensing methods were examined by running the calcium mobilization assay with cells plated with a MicroFlo Select dispenser; those results were compared to results obtained with manually plated cells. Further, compound dispense heights and speeds were optimized on the FLIPR Tetra to ensure proper dispensing without disturbing the cell monolayer. Additionally, we tested the effect of a no-wash protocol in which the calcium dye is added directly to the growth media containing the cells, thus making the assay procedure more amenable to HTS. No adverse effects of the no-wash protocol were observed; therefore, all subsequent assays in 384-well format were conducted in this manner. Using the fully optimized assay, we generated a Z’-factor [19] by running at least two 384-well plates containing positive ((1*R*,2*R*)-2-PCCA) and negative (2% DMSO/assay buffer) controls each day for 4 days. Although all of our efforts thus far used 1% DMSO, we needed to increase the amount to 2% in order to accommodate a final screening concentration of 20 μM. Representative data from one plate are shown in Fig. 4. On this plate, the average 10 μM (1*R*,2*R*)-2-PCCA signal was 1830 RFU with a standard deviation of 119 RFU and the average vehicle signal was 199 RFU with a standard deviation of 24 RFU; the Z’-factor was 0.74. The average Z’-factor ± SD from all the experiments was calculated to be 0.72 ± 0.07. A Z’-factor > 0.5 is appropriate for a HTS assay; therefore, our assay is deemed to be highly robust and reproducible, and hence suitable for HTS applications.

### Screening of a pilot neurotransmitter library

**Fig. 5:**
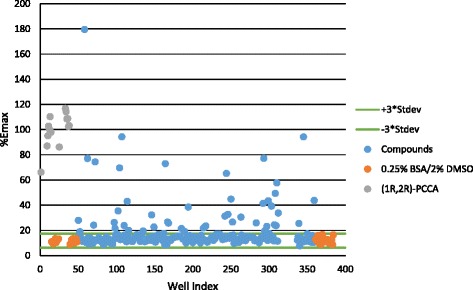
Scatter plot of screening a pilot neurotransmitter library. The primary screen was conducted as described in the Methods. Each data point represents a single well on a 384-well plate. (1*R*,2*R*)-2-PCCA and 0.25% BSA/2% DMSO (vehicle) are included as controls

A small neurotransmitter library (228 compounds, Enzo Life Sciences) was screened using the automated, miniaturized PPLS-HA-GPR88-Gα_qi5_-CHO calcium mobilization assay. All compounds were screened for agonist activity at 20 μM final concentration using (1*R*,2*R*)-2-PCCA as a positive control. Agonist screening data were collected and converted to % (1*R*,2*R*)-2-PCCA E_max_. Screening of the library yielded 10 compounds that increased fluorescence to >50% of the (1*R*,2*R*)-2-PCCA control (Fig. 5). However, a counter-screen against parental Gα_qi5_ cells suggested that the hits were false positives due to nonspecific mobilization of intracellular calcium.

## Discussion

GPCRs have attracted significant attention for drug discovery due to their numerous physiological and pathological roles in mediating cellular responses to hormones and neurotransmitters [21]. Within the large GPCR family, 140 GPCRs, excluding olfactory receptors, are classified as orphan receptors because their endogenous ligands are still unknown [22]. GPR88 is one of these orphan GPCRs that has recently attracted considerable interest because of its linkage to a number of basal ganglia-associated disorders. Despite the emerging pharmacological implications, the detailed functions of GPR88 are still largely unknown due to the lack of potent and selective ligands appropriate for CNS investigation. The availability of a simple, rapid and robust assay to monitor GPR88 activity would expedite the search for novel GPR88 ligands. Thus, the goal of this project was to develop a validated HTS assay for GPR88.

HTS campaigns for orphan GPCRs utilizing the measurement of intracellular calcium mobilization with FLIPR is a cost-effective, robust, and reproducible functional assay. Because calcium mobilization occurs after activation of Gα_q_-coupled receptors and not all receptors couple to Gα_q_, including GPR88 [14], a group of promiscuous Gα_q_ proteins was developed to enable GPCRs to mobilize calcium. Gα_q16_ is the most widely used promiscuous Gα_q_ protein; [23] however, not all receptors couple effectively to Gα_q16_ [24]. To circumvent this issue, the promiscuous Gα_qi5_ protein was created to more effectively couple Gα_i_-coupled receptors to Gα_q_ proteins by replacing the five Gα_q_ C-terminal amino acids with those from the Gα_i_ protein, thus allowing the Gα_i_-coupled receptor to respond to an agonist via elevation of intracellular calcium [25, 26]. In order to develop a sensitive and reliable assay applicable for HTS, we tested different cell systems and initially transfected GPR88 in Gα_q16_ and Gα_qi5_ CHO cells to determine which Gα_q_ protein would couple with GPR88 to induce calcium mobilization. Interestingly, we found that the GPR88 receptor agonist (1*R*,2*R*)-2-PCCA did not elicit a GPR88-specific response in the Gα_q16_ system, but did elicit a specific response in the Gα_qi5_ system (Fig. 1). In view of these findings, we created a stable PPLS-HA-GPR88-Gα_qi5_ CHO cell line for the calcium mobilization assay. The PPLS has been reported to enhance surface expression of a number of Gα_i_-coupled GPCRs [20, 27, 28], and therefore was used to promote GPR88 expression on the plasma membrane. In the stable PPLS-HA-GPR88-Gα_qi5_ CHO cells, (1*R*,2*R*)-2-PCCA had a robust signal with an EC_50_ = 468 nM. Although the response to (1*R*,2*R*)-PCCA was potent in our calcium mobilization assay, the concentration-response curve did not reach a top plateau. We believe this is due to the engineered G protein coupling present in the system as (1*R*,2*R*)-PCCA is a highly potent agonist that reaches a top plateau in our secondary cAMP assay [14, 16].

To further validate our stable GPR88 cell line, a number of (1*R*,2*R*)-2-PCCA analogues were tested to characterize their responses at GPR88 (Table 1). The phenyl analogue **1b** was approximately 2.5-fold less potent than (1*R*,2*R*)-2-PCCA. A large alkyl group such as cyclohexyl (**1c**, EC_50_ = 794 nM) was well tolerated at the 4’-position of the biphenyl moiety. However, substitution with an amide group (**1d**) resulted in a loss of activity. In an examination of the amino alkyl side chain’s effect on potency, the leucine derivative **2a** had an EC_50_ value similar to the isoleucine analogue **1b** (1320 nM vs 1260 nM). Changing the *S*-configuration of the amino group to the *R*-configuration (**2b**) led to a 2-fold loss of potency. Moreover, the isohexyl compound **2c** was completely inactive, indicating the amino group is critical for activity, possibly because it forms a hydrogen bonding interaction with the receptor binding site. Finally, the (*R*,*R*)-configuration of the cyclopropyl is also critical for activity as its (*S*,*S*)-isomer resulted in an inactive compound **3**. In general, the SAR trends presented herein agree with the data reported from the past studies [14–16]. In addition, compounds were also tested in the parental Gα_qi5_ CHO cells and exhibited no activity at 10 μM final concentration (see Additional file 1: Figure S1). Taken together, these studies indicated that coupling to Gα_qi5_ did not affect functional activation of GPR88 and that the calcium mobilization assay was capable of detecting GPR88 agonists. The 96-well assay was then optimized and adapted for HTS applications by miniaturizing to 384-well and testing multiple variables (cell density, solvent tolerance, and no-wash protocol). The assay was deemed robust and reproducible with a Z’-factor of 0.72 ± 0.07, and performed well with the no-wash protocol in buffer containing DMSO at a final concentration up to 2%.

GPR88 is most closely related to the biogenic amine receptors, and has the highest sequence homology with the 5-HT_1d_ receptor and the β_3_ adrenergic receptor (27 and 21% identity, respectively) [6]. Chemogenomic analysis, based on the alignment of 30 critical residues predicted to line the binding cavity of GPCRs, clustered GPR88 with metabotropic glutamate and GABA_B_ receptors, suggesting they may bind to structurally related ligands [29]. Accordingly, a pilot neurotransmitter library, including natural and synthesized ligands of adrenergic, serotonergic and GABA_B_ receptors, was screened and yielded 10 hits, but none of the hits were confirmed as GPR88 agonists in follow-up assays. A larger HTS campaign in the future using a more diverse set of compounds that occupy different chemical spaces might lead to the discovery of novel GPR88 agonists.

To the best of our knowledge, this is the first report describing the development and validation of a GPR88 HTS assay. The only other scientific document outlining such efforts is a university thesis available online through the Leiden University Repository [30]. This report describes efforts in the development of a calcium mobilization assay for GPR88 using the promiscuous Gα_q_ proteins (Gα_q16_ or Gα_qi5_) in HEK293 cells; however, no known GPR88 ligands were used to validate the assay. Furthermore, the report’s stable GPR88-Gα_qi5_-HEK293 cells suffered loss of GPR88 expression over time, as only 40% of the cell population was positive for GPR88 expression 3 weeks after fluorescent automated cell sorting (FACS) enrichment [30]. Interestingly, during the course of developing our GPR88 assays, we independently observed that HEK293 cells physically appeared unhealthy shortly after transfection with GPR88 and hence produced deteriorated responses to the agonist (1*R*,2*R*)-2-PCCA over time. Due to those complications, we opted to use GPR88-CHO cells for our HTS assay.

Recent efforts in our laboratory have focused on the development of cAMP assays for the orphan GPR88 [14, 16, 18]. While the cAMP assay is a sensitive assay platform that measures GPR88 activation, using this platform for HTS has one major drawback. Because the cAMP assay is an endpoint assay, only one mode of activity can be measured in this format in a single screen. Our laboratory is interested in identifying multiple types of ligands for GPR88, so developing a HTS assay that can measure more than one mode of activity at a time was highly desirable. Our laboratory has developed a general FLIPR-based calcium mobilization protocol which allows for both agonist and antagonist screening with a single assay plate, thereby cutting screening time in half.

## Conclusions

In conclusion, we have developed a CHO-Gα_qi5_-GPR88 cell-based calcium mobilization assay for the orphan receptor GPR88. The assay was validated by the SAR study of a small group of (1*R*,2*R*)-2-PCCA analogues, and was deemed robust and suitable for HTS applications. Although our work thus far has been aimed at the identification of GPR88 receptor agonists, in theory, this calcium mobilization assay can be used to identify other ligands as well, including competitive and noncompetitive antagonists, inverse agonists and allosteric modulators. These ligands will serve as valuable tools to probe signaling mechanisms and in vivo functions of GPR88, and could expedite development of novel therapies for diseases potentially mediated by GPR88. Such efforts are currently under investigation in our laboratory.

## Additional file

Additional file 1: Development and validation of a high-throughput calcium mobilization assay for the orphan receptor GPR88. (DOC 316 kb)

## Abbreviations

cAMP: Cyclic AMP
CHO: Chinese hamster ovary
CNS: Central nervous system
DMSO: Dimethyl sulfoxide
FLIPR: Fluorescent imaging plate reader
GPCR: G protein-coupled receptor
HA: Influenza hemagglutinin
HEK: Human embryonic kidney
HTS: High-throughput screen
MSNs: Medium spiny neurons
PPLS: Pre-prolactin leader sequence
