# Supplementary material for: Development and validation of a high-throughput calcium mobilization assay for the orphan receptor GPR88
Source: J Biomed Sci. 2017 Mar 27;24:23. doi: 10.1186/s12929-017-0330-3 (PMC5369193; doi:10.1186/s12929-017-0330-3)
Supplement: Additional file 1: — Development and validation of a high-throughput calcium mobilization assay for the orphan receptor GPR88. (DOC 316 kb) [file 12929_2017_330_MOESM1_ESM.doc]

**Development and Validation of a High-Throughput Calcium Mobilization Assay for the Orphan Receptor GPR88**

*Ann M. Decker, Elaine A. Gay, Kelly M. Mathews, Taylor C. Rosa, Tiffany L. Langston, Rangan Maitra, and Chunyang Jin ^*^*

Center for Drug Discovery, Research Triangle Institute, Research Triangle Park, North Carolina 27709, USA

**Additional file 1**

Characterization of compounds **1a−d**, **2a−c** and **3**

**
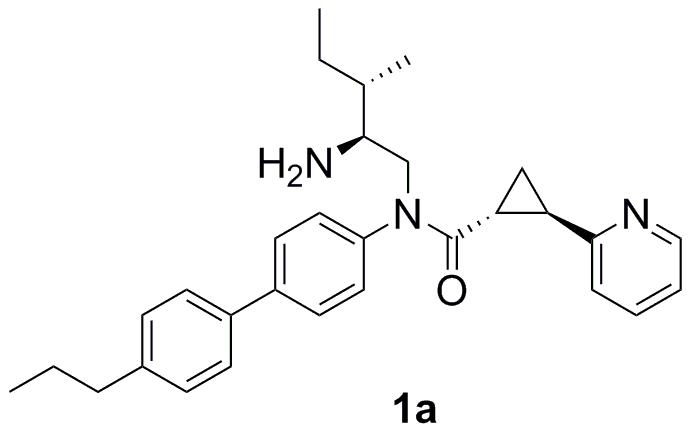
**

*(1R,2R)-2-(Pyridin-2-yl)cyclopropanecarboxylic Acid [(2S,3S)-2-Amino-3-methylpentyl]-(4’-propylbiphenyl-4-yl)amide (****1a****).* ^1^H NMR (300 MHz; CD_3_OD) δ 8.58 (d, *J* = 6.0 Hz, 1H), 8.28 (t, *J* = 9.0 Hz, 1H), 7.78−7.52 (m, 6H), 7.51 (d, *J* = 9.0 Hz, 2H), 7.27 (d, *J* = 9.0 Hz, 2H), 4.29 (dd, *J* = 15.0, 9.0 Hz, 1H), 3.83 (d, *J* = 12.0 Hz, 1H), 3.48−3.38 (m, 1H), 3.01−2.90 (m, 1H), 2.62 (t, *J* = 7.5 Hz, 2H), 2.22−2.12 (m, 1H), 2.02−1.92 (m, 1H), 1.90−1.75 (m, 1H), 1.75−1.60 (m, 3H), 1.50−1.35 (m, 1H), 1.35−1.15 (m, 1H), 1.05−0.90 (m, 6H), 0.86 (t, *J* = 6.0 Hz, 3H); ^13^C NMR (75 MHz; CD_3_OD) δ 173.4, 157.6, 146.6, 143.9, 143.3, 142.9, 141.6, 138.2, 130.2, 129.8, 129.5, 127.9, 125.5, 125.1, 56.6, 50.9, 38.7, 37.2, 27.4, 26.5, 25.7, 25.2, 18.1, 14.2, 14.2, 11.7; HRMS (ESI) calcd. for C_30_H_37_N_3_O [M + H]^+^: 456.3009. Found: 456.3023.

**
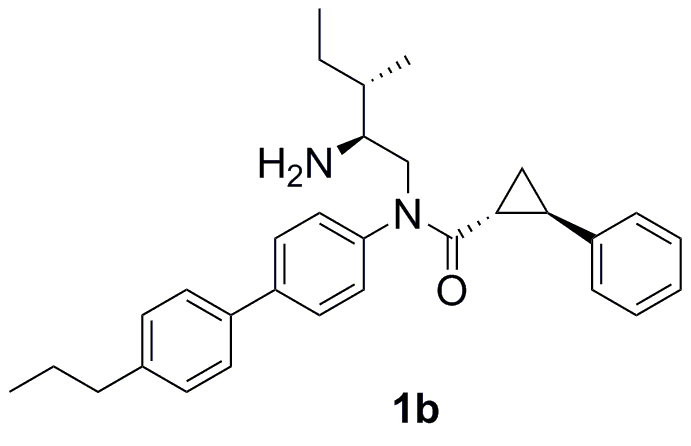
**

*(1R,2R)-2-Phenyl-1-cyclopropanecarboxylic Acid [(2S,3S)-2-Amino-3-methylpentyl]-(4’-propylbiphenyl-4-yl)amide (****1b****).* ^1^H NMR (300 MHz; CD_3_OD) δ 7.65 (br d, *J* = 6.0 Hz, 2H), 7.56−7.38 (m, 4H), 7.26 (d, *J* = 9.0 Hz, 2H), 7.20−7.06 (m, 3H), 6.93 (d, *J* = 9.0 Hz, 2H), 4.38−4.22 (m, 1H), 3.83−3.60 (m, 1H), 3.44−3.33 (m, 1H), 2.62 (t, *J* = 7.5 Hz, 2H), 2.54−2.42 (m, 1H), 1.85−1.60 (m, 4H), 1.50−1.16 (m, 4H), 1.03−0.93 (m, 6H), 0.87 (t, *J* = 6.0 Hz, 3H); ^13^C NMR (75 MHz; CD_3_OD) δ 176.0, 143.8, 142.7, 142.0, 141.5, 138.4, 130.3, 129.8, 129.6, 129.5, 128.0, 127.5, 127.2, 57.0, 50.7, 38.7, 37.3, 28.1, 26.5, 26.0, 25.7, 17.9, 14.4, 14.2, 11.9; HRMS (ESI) calcd. for C_31_H_38_N_2_O [M + H]^+^: 455.3057. Found: 455.3061.


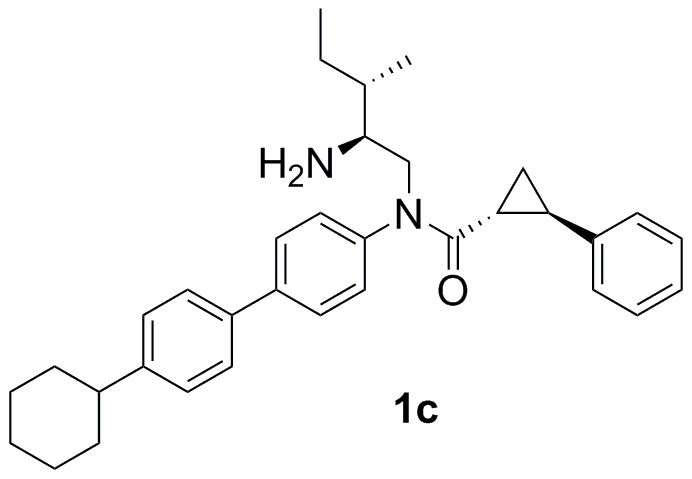


*(1R,2R)-2-Phenyl-1-cyclopropanecarboxylic Acid [(2S,3S)-2-Amino-3-methylpentyl]-(4’-cyclopropylbiphenyl-4-yl)amide (****1c****).* ^1^H NMR (300 MHz; CD_3_OD) δ 7.65 (d, *J* = 6.0 Hz, 2H), 7.58−7.40 (m, 4H), 7.31 (d, *J* = 9.0 Hz, 2H), 7.22−7.08 (m, 3H), 6.94 (d, *J* = 6.0 Hz, 2H), 4.38−4.22 (m, 1H), 3.78 (dd, *J* = 15.0, 3.0 Hz, 1H), 3.42−3.30 (m, 1H), 2.60−2.40 (m, 2H), 1.98−1.60 (m, 8H), 1.58−1.18 (m, 8H), 0.99 (d, *J* = 6.0 Hz, 3H), 0.88 (t, *J* = 7.5 Hz, 3H); ^13^C NMR (75 MHz; CD_3_OD) δ 176.0, 149.2, 142.7, 142.0, 141.4, 138.4, 129.7, 129.5, 129.4, 128.5, 128.0, 127.5, 127.1, 56.9, 50.8, 45.6, 37.2, 35.7, 28.0, 27.3, 26.5, 25.9, 25.7, 17.9, 14.3, 11.8; HRMS (ESI) calcd. for C_34_H_42_N_2_O [M + H]^+^: 495.3370. Found: 495.3388.


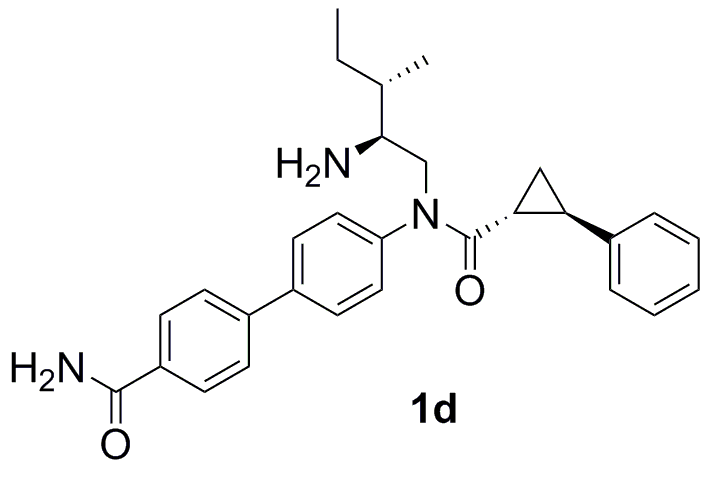


*(1R,2R)-2-Phenyl-1-cyclopropanecarboxylic Acid [(2S,3S)-2-Amino-3-methylpentyl]-(4’-carbamoylbiphenyl-4-yl)amide (****1d****).* ^1^H NMR (300 MHz; CD_3_OD) δ 7.92−7.80 (m, 2H), 7.70−7.50 (m, 4H), 7.50−7.30 (m, 2H), 7.12−6.95 (m, 3H), 6.90−6.88 (m, 2H), 4.30−4.15 (m, 1H), 3.78−3.62 (m, 1H), 3.35−3.21 (m, 1H), 2.46−2.30 (m, 1H), 1.80−1.56 (m, 4H), 1.40−1.25 (m, 2H), 0.78 (d, J = 6.0 Hz, 3H), 0.58 (t, *J* = 6.0 Hz, 3H); ^13^C NMR (75 MHz; CD_3_OD) δ 175.9, 169.8, 144.3, 143.0, 141.5, 141.4, 130.0, 129.9, 129.5, 128.2, 127.5, 127.1, 57.0, 50.8, 37.3, 28.1, 26.5, 26.1, 17.9, 14.3, 11.9; HRMS (ESI) calcd. for C_31_H_38_N_2_O [M + H]^+^: 455.2573. Found: 455.2581.


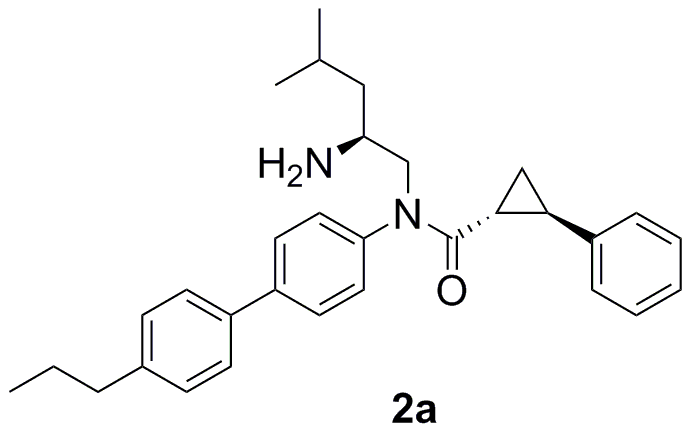


*(1R,2R)-2-Phenyl-1-cyclopropanecarboxylic Acid [(2S)-2-Amino-4-methylpentyl]-(4’-propylbiphenyl-4-yl)amide (****2a****).* ^1^H NMR (300 MHz; CD_3_OD) δ 7.53 (d, *J* = 6.0 Hz, 2H), 7.42−7.28 (m, 4H), 7.16 (d, *J* = 9.0 Hz, 2H), 7.12−6.99 (m, 3H), 6.86 (d, *J* = 9.0 Hz, 2H), 4.15−4.00 (m, 1H), 3.84 (dd, *J* = 15.0, 3.0 Hz, 1H), 3.42−3.30 (m, 1H), 2.53 (t, *J* = 7.5 Hz, 2H), 2.44−2.34 (m, 1H), 1.65−1.10 (m, 8H), 0.86 (t, *J* = 7.5 Hz, 3H), 0.76 (d, *J* = 6.0 Hz, 3H), 0.74 (d, *J* = 6.0 Hz, 3H); ^13^C NMR (75 MHz; CD_3_OD) δ 176.1, 143.8, 142.6, 142.3, 141.4, 138.3, 130.2, 129.5, 129.4, 129.3, 127.9, 127.5, 127.0, 53.0, 51.5, 40.8, 38.7, 28.1, 25.9, 25.7, 25.5, 22.8, 22.2, 17.6, 14.1; HRMS (ESI) calcd. for C_31_H_38_N_2_O [M + H]^+^: 455.3057. Found: 455.3071.


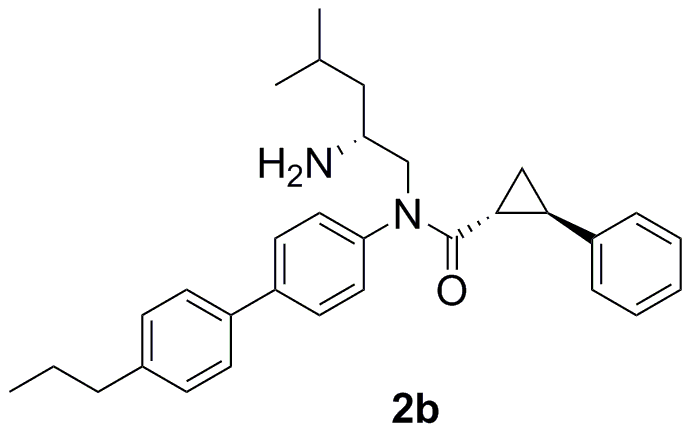


*(1R,2R)-2-Phenyl-1-cyclopropanecarboxylic Acid [(2R)-2-Amino-4-methylpentyl]-(4’-propylbiphenyl-4-yl)amide (****2b****).* ^1^H NMR (300 MHz; CD_3_OD) δ 7.52 (d, *J* = 9.0 Hz, 2H), 7.41−7.30 (m, 4H), 7.15 (d, *J* = 6.0 Hz, 2H), 7.10−6.98 (m, 3H), 6.86 (d, *J* = 6.0 Hz, 2H), 4.16−4.00 (m, 1H), 3.81−3.70 (m, 1H), 3.40−3.35 (m, 1H), 2.50 (t, *J* = 7.5 Hz, 2H), 2.50−2.48 (m, 1H), 1.80−1.20 (m, 8H), 0.96 (t, *J* = 7.5 Hz, 3H), 0.89 (d, *J* = 6.0 Hz, 3H), 0.85 (d, *J* = 6.0 Hz, 3H); ^13^C NMR (75 MHz; CD_3_OD) δ 176.1, 143.8, 142.6, 142.1, 141.4, 138.3, 130.2, 129.4, 129.3, 127.9, 127.4, 127.1, 53.3, 51.3, 40.8, 38.7, 28.0, 25.9, 25.7, 25.6, 22.8, 22.2, 17.6, 14.1; HRMS (ESI) calcd. for C_31_H_38_N_2_O [M + H]^+^: 455.3057. Found: 455.3070.


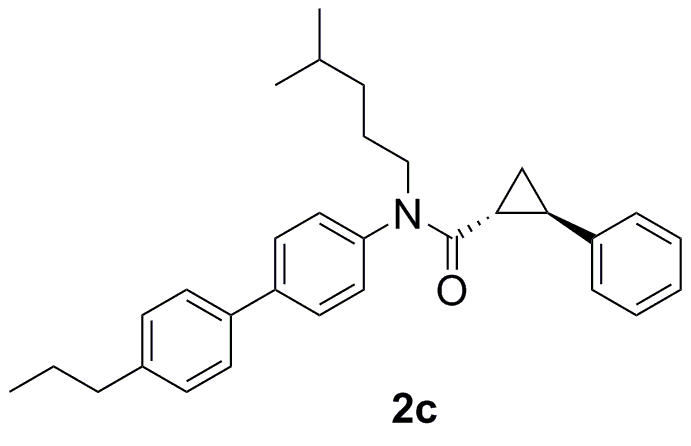


*(1R,2R)-2-Phenyl-1-cyclopropanecarboxylic Acid [4-Methylpentyl]-(4’-propylbiphenyl-4-yl)amide (****2c****).* ^1^H NMR (300 MHz; CD_3_OD) δ 7.53 (d, *J* = 9.0 Hz, 2H), 7.48 (d, *J* = 9.0 Hz, 2H), 7.30−7.10 (m, 7H), 6.94 (d, *J* = 6.0 Hz, 2H), 3.90−3.66 (m, 2H), 2.63 (t, *J* = 7.5 Hz, 2H), 2.60−2.50 (m, 1H), 1.78−1.45 (m, 7H), 1.30−1.05 (m, 3H), 0.97 (t, *J* = 7.5 Hz, 3H), 0.85 (d, *J* = 6.0 Hz, 6H); ^13^C NMR (75 MHz; CD_3_OD) δ 171.7, 142.3, 141.4, 140.8, 140.3, 137.3, 129.0, 128.5, 128.3, 127.8, 126.8, 126.3, 126.1, 49.8, 37.7, 35.9, 27.8, 26.3, 25.8, 24.5, 24.2, 22.6, 17.1, 13.8; HRMS (ESI) calcd. for C_31_H_38_N_2_O [M + H]^+^: 440.2948. Found: 440.2961.


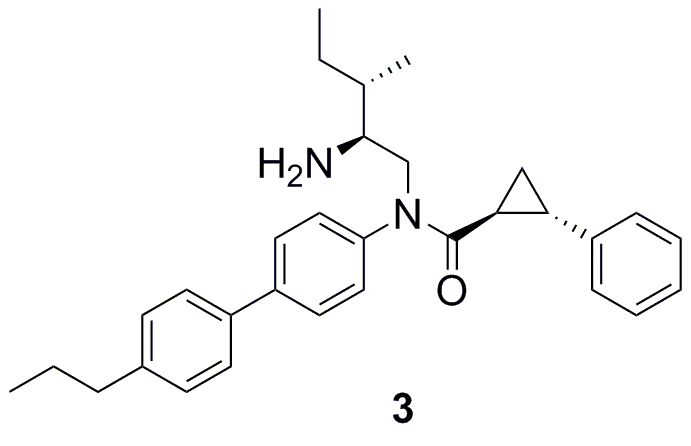


*(1S,2S)-2-Phenyl-1-cyclopropanecarboxylic Acid [(2S,3S)-2-Amino-3-methylpentyl]-(4’-propylbiphenyl-4-yl)amide (****3****).* ^1^H NMR (300 MHz; CD_3_OD) δ 7.61 (br d, *J* = 6.0 Hz, 2H), 7.50−7.40 (m, 4H), 7.25 (d, *J* = 6.0 Hz, 2H), 7.22−7.08 (m, 3H), 6.98 (d, *J* = 9.0 Hz, 2H), 4.33 (dd, *J* = 15.0, 9.0 Hz, 1H), 3.75 (dd, *J* = 15.0, 3.0 Hz, 1H), 3.38−3.30 (m, 1H), 2.62 (t, *J* = 7.5 Hz, 2H), 2.57−2.50 (m, 1H), 1.71−1.60 (m, 4H), 1.45−1.15 (m, 4H), 1.02−0.92 (m, 6H), 0.80 (t, *J* = 9.0 Hz, 3H); ^13^C NMR (75 MHz; CD_3_OD) δ 176.1, 143.8, 142.6, 141.8, 141.5, 138.4, 130.3, 129.7, 129.5, 129.4, 128.0, 127.5, 127.2, 56.8, 50.7, 38.8, 37.4, 28.2, 26.6, 26.1, 25.8, 17.5, 14.2 (two overlap carbon peaks), 11.9; HRMS (ESI) calcd. for C_31_H_38_N_2_O [M + H]^+^: 455.3057. Found: 455.3062.

**Figure S1.** Activity of (1*R*,2*R*)-2-PCCA and analogues in counter-screen. Compounds were screened for activity at 10 µM final concentration in parental Gα_qi5_-CHO cells (□). The response at 10 µM final in stable PPLS-HA-GPR88-Gα_qi5_-CHO cells (■) is provided as a comparison. Representative data are shown and bars are mean ± SD of duplicate determinations.
